# Aspen Increase Soil Moisture, Nutrients, Organic Matter and Respiration in Rocky Mountain Forest Communities

**DOI:** 10.1371/journal.pone.0052369

**Published:** 2012-12-17

**Authors:** Joshua R. Buck, Samuel B. St. Clair

**Affiliations:** Department of Plant and Wildlife Sciences, Brigham Young University, Provo, Utah, United States of America; DOE Pacific Northwest National Laboratory, United States of America

## Abstract

Development and change in forest communities are strongly influenced by plant-soil interactions. The primary objective of this paper was to identify how forest soil characteristics vary along gradients of forest community composition in aspen-conifer forests to better understand the relationship between forest vegetation characteristics and soil processes. The study was conducted on the Fishlake National Forest, Utah, USA. Soil measurements were collected in adjacent forest stands that were characterized as aspen dominated, mixed, conifer dominated or open meadow, which includes the range of vegetation conditions that exist in seral aspen forests. Soil chemistry, moisture content, respiration, and temperature were measured. There was a consistent trend in which aspen stands demonstrated higher mean soil nutrient concentrations than mixed and conifer dominated stands and meadows. Specifically, total N, NO_3_ and NH_4_ were nearly two-fold higher in soil underneath aspen dominated stands. Soil moisture was significantly higher in aspen stands and meadows in early summer but converged to similar levels as those found in mixed and conifer dominated stands in late summer. Soil respiration was significantly higher in aspen stands than conifer stands or meadows throughout the summer. These results suggest that changes in disturbance regimes or climate scenarios that favor conifer expansion or loss of aspen will decrease soil resource availability, which is likely to have important feedbacks on plant community development.

## Introduction

Forest community types are often associated with specific soil classes, and soil chemistry and texture have important influences on forest function [1], [2]. Plant-soil interactions in turn have important feedbacks on soil traits [3] that contribute to patterns of plant community development over time [4], [5]. As plant communities change, corresponding shifts in stand productivity and architecture, litter quantity and quality, root traits and microbial activity can alter soil moisture status, decomposition rates, nutrient cycling, and soil-atmosphere gas fluxes that are important controls of forest ecosystem function [6], [7].

*Populus tremuloides* (Michx) is a keystone tree species in subalpine and boreal forests of North America. In mid elevation forests of the Rocky Mountains, aspen are often associated with conifers in mixed forest communities that develop under cycles of secondary succession [8]. Each cycle begins with a disturbance event, typically fire that removes the overstory stand and releases the aspen root system from apical dominance, usually resulting in root suckering that forms the foundation for re-establishing the forest community [9]. In time, aspen facilitate the establishment of conifer seedlings that grow rapidly and expand in size resulting in competitive interactions that can promote conifer dominance and aspen mortality until fire returns and initiates a new succession cycle [10]. Secondary succession in aspen-conifer forests changes both overstory and understory plant community characteristics through time [11]. Aspen stands tend to have higher biodiversity and productivity than both the forest meadow into which they expand, and conifer dominated stands that in the absence of disturbance replace them [12]. These shifts in plant community characteristics can alter soil characteristics and initiate a sequence of plant-soil interactions and feedbacks [13], [14], [15], [16]. For example, there is evidence that aspen accumulates more snowpack than open meadows or conifer stands, which likely has large impacts on the hydrological and developmental characteristics of these community types [17]. While differences in various soil characteristics have been compared under aspen versus conifer dominated stands in boreal forests [18], [19], [20], few studies have examined how soil traits vary across gradients of forest community composition (meadow→aspen dominant→mixed→ conifer dominant).

The characteristics and timing of disturbance is a key driver of successional outcomes in plant communities [21]. Fire suppression [22] and climate conditions [23] can alter fire intervals in aspen-conifer forests [24], [25]. Longer fire intervals promote late successional conditions that increase conifer abundance in aspen-conifer forests [25], [26]. Aspen regeneration tends to decrease under conifer dominance [8], [9] a response that is partially driven by changes in soil chemistry [10]. We are interested in understanding plant-soil interactions and feedbacks in mixed montane forests (which are much more poorly studied than boreal aspen forests), and how differences in overstory forest composition correspond to soil characteristics. This will provide a framework for understanding how shifts in stand composition, based on changing disturbance regimes are likely to affect plant-soil relations that underlie forest community development. We hypothesize that soil resource availability and activity (as measured by soil respiration) are relatively low in meadows, increase under aspen dominated stands and then decrease with greater conifer abundance.

## Methods

### Field sites and experimental design

**Figure 1:**
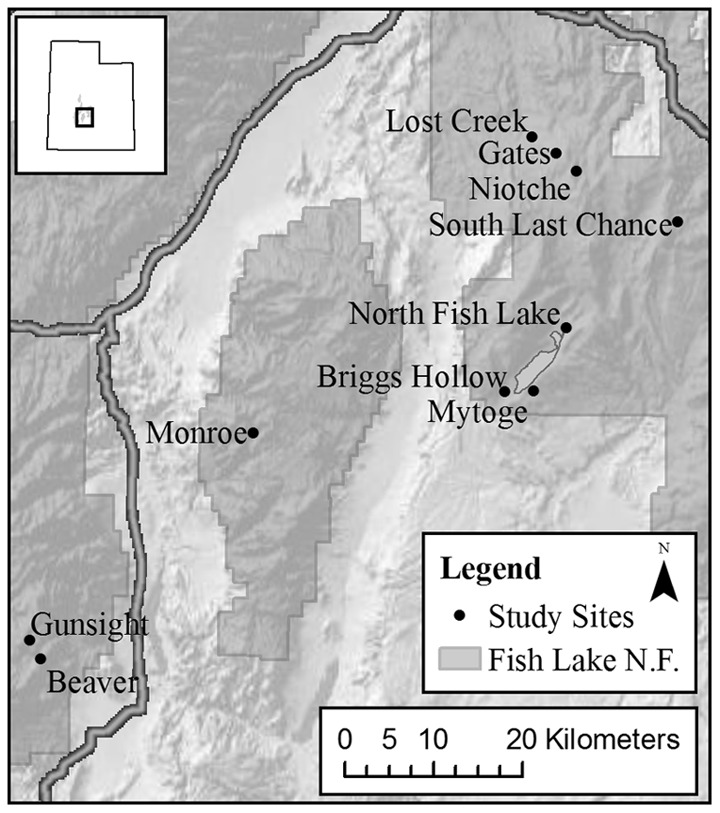
Map of the study sites on the Fishlake National Forest. Inset map of the state of Utah, USA with the study area outlined with the coordinates of the map center at: 38°30′32.26′′N and 111°52′55.94′′ W. Map was created using ArcGIS ArcMap v9.3.

This study was conducted at ten field sites across the Fishlake National Forest in central Utah (Fig. 1). Each of the ten sites had four adjacent stands (<25 m distance) that varied in overstory composition as follows: predominantly aspen (>75% aspen stems), equal mix of aspen and conifer (∼50% aspen and conifer stems), predominantly conifer, which was dominated by subalpine fir but also included Engelmann spruce (*Picea engelmannii* Parry ex Engelm) and Douglas-fir (*Pseudotsuga menziesii* Carriere) (>75% conifer stems), and open meadow without trees that was immediately adjacent to the forest edge. Differences in canopy composition at each field site were representative of stages in the pathway of secondary succession that is initiated by disturbance, followed by aspen establishment and ending with conifer dominance. Tree composition and density in each stand type were calculated using the point quarter method along a 50 meter transect [27]. The percentage of aspen to conifer in the aspen, mixed and conifer stands were 90∶10, 51∶49 and 24∶76. Average stand densities for the aspen, mixed, and conifer stands were: 2228±472, 2806±428 and 1978±548 stems ha^−1^ (included trees with diameter ≥8 cm). Basal tree area for the aspen, mixed and conifer stands were: 58±12, 76±13 and 59±18 m^2^ ha^−1^. Shrubs (*Symmphoricarpos, Amelanchier*), grasses (*Agropyron, Bromus*) and forbs (*Achillea, Vicia*) were common in the vascular plant understory with plant density and cover being greatest under aspen dominated stands. Adjacent meadow consisted of mixed grass-forbs and low density shrubs, particularly sagebrush. Site elevations ranged from 2700m to 3000m and stand slopes varied from 6–23 degrees. While aspect, elevation and slope differed between sites, they did not vary significantly among stand types. Because stands were adjacent and occurred on similar aspects, it is assumed that they experienced similar temperature and precipitation patterns.

Soil samples for nutrient analysis were collected from each of the four stand types at seven field sites in July of 2008, with an additional three sites sampled in August of 2011. Permits for soil sampling were obtained from the Fishlake National Forest. Soil profiles were dug and soil samples were collected at three points along a 50 meter transect in each of the three stand types, and in an open meadow immediately adjacent to the stands. Two soil samples were collected from each pit. The first, termed the OA fraction, was collected from the soil surface (including the O horizon) to the A–B soil horizon boundary (typically 0–10 cm in depth). The second soil sample was collected from the B-horizon (typically 10–25 cm in depth). The samples were placed in plastic bags and were transported back to the lab in a cooler.

### Soil chemistry

Soil samples were analyzed for total nitrogen, ammonium-nitrogen, nitrate-nitrogen, carbon, organic matter, pH, and mineral nutrient concentrations (P, K, Ca, Mg, Fe, Mn, Cu Zn). Soil texture was measured by the hydrometer method [28]. Percent nitrogen and carbon were determined using a CN analyzer (Truspec CN Determinator, LECO Cooperation, St. Joseph, Michigan, USA). Ammonium and nitrate concentrations were determined colorimetrically using a rapid flow analyzer (Lachat QuickChem 8500, Lachat Instruments, Loveland, CO, USA). Percent organic matter was measured using the dichromate oxidation method [29]. Soil pH was assessed using the saturated paste method with a pH meter. Bioavailable phosphorus and potassium concentrations were measured by a sodium bicarbonate extraction [30]. Exchangeable Ca, Mg, K, and Na were extracted with ammonium acetate and Cu, Zn, Fe and Mn with DTPA [31], [32]. Soil cation concentrations were measured using inductively coupled plasma spectroscopy (Iris Intrepid II XSP, Thermo Electron Cooperation, Waltham, MA, USA).

### Soil moisture content

Measurements of soil moisture content were taken at three points along the same 50m transects in each stand type using a Field Scout 100 time-domain reflectometry (TDR) probe with 12 cm rods (Spectrum Technologies Inc., Plainfield, IL, USA). Three measurements were taken at each measurement point and averaged together. Soil moisture measurements were taken June 8–11, July 20–22, and August 24–25, 2009. Values were recorded as percent volumetric water content (%VWC).

### Soil respiration (CO_2_ efflux) and temperature

Soil respiration was measured using a gas exchange system with a soil CO_2_ flux chamber (Li-Cor 6400, Li-Cor Biosciences, Lincoln, NE, USA) at three points along the same 50 m transects. PVC collars (10 cm tall and 10 cm diameter) inserted 5 cm into the soil surface were used to create a standard sampling volume for each measurement. Readings at each site were taken within the same hour and the order in which measurement were taken was randomized within sites. Soil temperature was measured simultaneously with CO_2_ efflux measurements using a soil temperature probe inserted 10 cm into the soil (Li-Cor 6400, Li-Cor Biosciences, Lincoln, NE, USA). Measurements were taken at the same time points and locations as soil moisture measurements during the summer of 2009.

### Statistical analysis

Differences in soil characteristics among stand types were tested using analysis of variance. In the ANOVA models, stand type was treated as a fixed effect with blocking across sites. Multiple comparisons among stand types were determined using a Tukey's adjusted t-test. Data were tested for normality and homogeneity of variance using Shapiro-Wilk W statistics and equal variance tests. Time-course measurements of soil moisture, CO_2_ efflux, and temperature were analyzed for stand type and time differences using a repeated measure ANOVA model. Statistical analysis was performed using JMP version 8.0.1 statistical software (SAS Institute, Cary, NC, USA).

## Results

### Soil chemistry

For the OA soil fraction, organic matter, C:N, total N, K, Fe, and Zn demonstrated statistically significant differences (*P*<0.05) between stand types in the ANOVA analysis, while NO_3_, P, and Mn showed slightly weaker stand effects (0.05>*P*≤0.075) (Table 1). Specifically, total N, NO_3_ and NH_4_ were nearly two-fold higher in aspen stands than mixed and conifer stands or meadow soils. Organic matter, total N, and Zn were greatest in aspen stands followed by mixed and conifer dominated stands and were lowest in meadows (Table 1). Potassium concentrations followed the same trend; however conifer stands had lower K than meadows. Conifer stands had the highest C:N ratio and Fe concentrations when compared to the other stand types (Table 1). Aspen stand soils had significantly higher total N and K, with a lower C:N ratio than conifer stands (Table 1).

**Table 1:** Soil chemistry data presented by stand type.

Soil: OA Horizon	pH	Organic Matter (%)	C:N	Total N (%)		NH_4_-N (µg/g)	NO_3_-N (µg/g)	P (µg/g)
Meadow	5.6±.12	5.6±1.7^b^	26.1±2.1^b^	0.20±0.04^b^		12.0±5.2	8.3±3.7	32.9±7.8
Aspen	5.6±.12	12.7±1.7^a^	25.0±2.1^b^	0.44±0.04^a^		28.5±5.2	19.5±3.7	61.3±7.8
Mixed	5.7±.12	9.6±1.7^ab^	30.3±2.1^ab^	0.29±0.04^ab^		19.0±5.2	7.8±3.7	52.9±7.8
Conifer	5.7±.12	8.8±1.7^ab^	35.5±2.1^a^	0.24±0.04^b^		15.5±5.2	7.7±3.7	57.5±7.8
F-value	0.09	3.10	4.95	5.63		1.87	2.5	2.66
P-value	0.96	0.033	0.004		0.002	0.145	0.068	0.056

Soil: OA Horizon	K (µg/g)	Ca (µg/g)	Mg (µg/g)	Fe (µg/g)	Mn (µg/g)	Cu (µg/g)	Zn (µg/g)
Meadow	342±48^b^	2872±325	239±36	99±10.7^b^	26±5.4	.85±.09	2.31±1.2^b^
Aspen	531±48^a^	3936±325	319±36	141±10.7^a^	43±5.4	1.1±.09	6.73±1.2^a^
Mixed	374±48^ab^	3723±325	258±36	133±10.7^ab^	45±5.4	.92±.09	3.31±1.2^ab^
Conifer	268±48^b^	3685±325	217±36	157±10.7^a^	38±5.4	1.0±.09	2.85±1.2^ab^
F-value	5.34	2.07	1.51	5.25	2.42	1.18	2.86
P-value	0.003	0.114	0.222	0.003	0.075	0.326	0.044

Mean and standard error ±1 SE presented. Superscript lettering represents differences in paired comparisons.

Soil chemistry in B horizon samples did not vary significantly between stand types (Fe and Zn: *P* = 0.17 and 0.11; all other nutrients with *P*-values >0.45). Soil texture across sites varied from a loam to sandy loam, but soil texture as assessed by the percent of sand, silt and clay did not differ significantly between stand types (Sand: *P* = 0.98, Silt: *P* = 0.95, Clay: *P* = 0.54).

### Soil temperature

**Figure 2:**
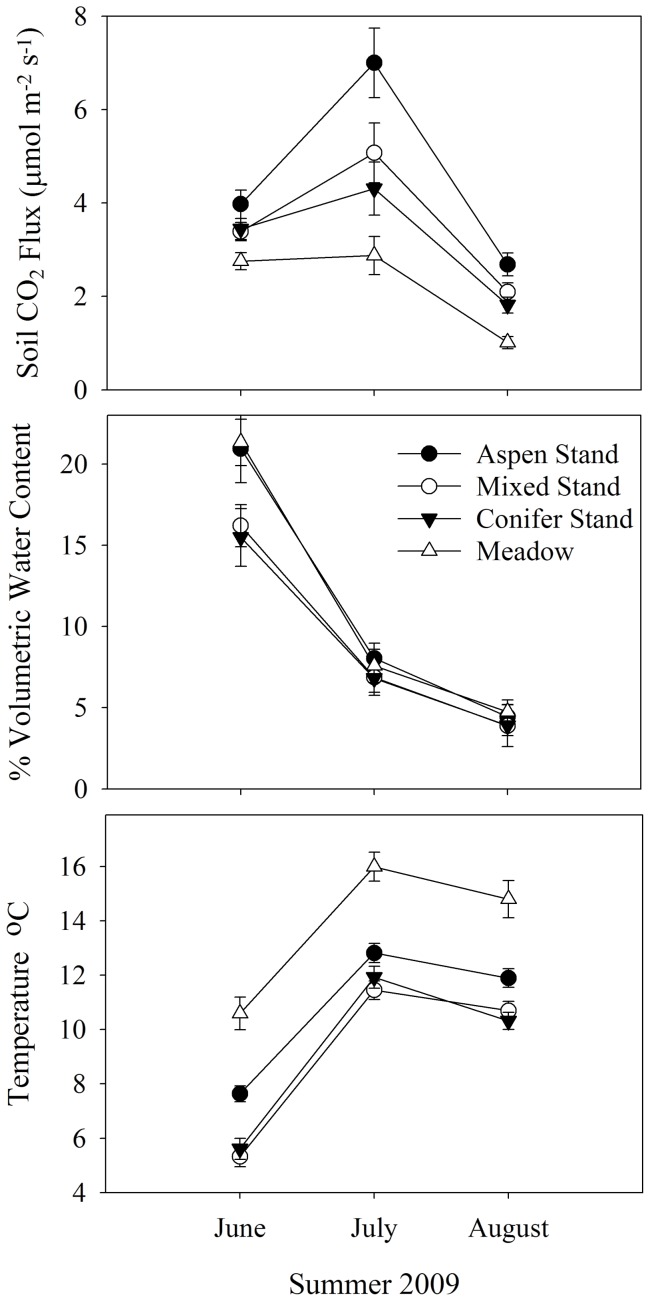
The influence of stand type on soil temperature, moisture and CO_2_ efflux over the summer of 2009. For soil temperature, the main effects in the repeated measures ANOVA model were significant but the interaction term was not: stand (*F*_3_,_80_  = 38, *P*<0.0001), time (*F F*_2_,_79_  = 94, *P*<0.0001), stand × time (*F*_6_,_158_  = 1.7307, *P*<0.1171). For soil moisture, stand type was not significant (*F*_3_,_80_  = 1.67, *P* = 0.17), but the main effect of time (*F*_2_,_79_  = 249, *P*<.0001) and the stand by time interaction were significant (*F*_6_,_158_  = 2.23, *P* = 0.04). For soil CO2 efflux both the main effects and the interaction term were statistically significant: stand type (*F*_3_,_80_  = 11.7, *P*<0.0001), time (*F*_2_,_79_  = 187, *P*<0.0001), stand × time (*F*_6_,_158_  = 2.31, *P* = 0.03). Means presented as symbols with error bars ±1 SE.

Meadows and aspen stands consistently had higher soil temperature (1–5°C, P<0.0001) over the course of the summer than mixed or conifer dominated stands (Figure 2). Changes in soil temperature across the summer were consistent across stand types; they increased approximately 5°C from early June to mid-July and then decreased by approximately 1°C by the end of summer (P<0.0001) (Figure 2).

### Soil moisture content

Soil moisture content decreased significantly for all stand types across the summer as indicated by the significant time effect in the repeated measures ANOVA model (Figure 2). Aspen stands and meadows had approximately 30% higher soil moisture content than mixed and conifer stands at the beginning of summer (*P* = 0.04), but mean values converged by mid-summer resulting in a significant stand x time interaction term (*P* = 0.04) (Figure 2).

### Soil respiration (CO_2_ efflux)

Soil respiration changed dynamically across the summer in all stand types as indicated by the strongly significant time variable (P<0.0001) in the repeated measures model (Figure 2). Across all four stand conditions, soil respiration increased from early June to mid-July where it peaked, and then decreased markedly from July to late August (Figure 2). Across the summer, aspen stands consistently had the highest soil respirations rates (aspen > mixed> conifer > meadow) (Figure 2). The significant interaction term (stand x time) was primarily the result of much greater differences in soil respiration rates between stands in mid-summer than was observed at the beginning or end of summer with aspen stands showing the strongest increase (175%) in July (Figure 2).

## Discussion

Plant-soil interactions play a critical role in structuring soil and plant community characteristics that underlie ecosystem function [33]. Plant-soil interactions can be reset through large scale disturbances, such as fire, that can result in shifts in soil microbial communities and changes in soil resource availability [34], [35]. The developmental patterns of plant communities in periods between disturbance events can influence soil characteristics that also feed back on plant community development [36]. We examined how differences in overstory stand composition in aspen-conifer forests correspond to forest soil properties. Although this is an observational and not a manipulative study, the data are consistent with our hypothesis in which soil resource availability and activity (respiration) increase from meadow to aspen dominated stands and then decreased with greater conifer abundance.

### Soil chemistry

There are multiple avenues for nutrient inputs from plants into soils, including: root exudates, root turnover, litter inputs, and stemflow [37], [38]. Differences in litter quality produced in aspen, meadow and conifer communities likely contribute to shifts in soil nutrient status that we observed across the stand composition gradients in our study. Foliar nitrogen content has been used to accurately predict soil nitrogen availability across differing forest stand types due to soil-plant feedbacks [39]. The litter of broadleaf species generally and aspen specifically tend to have higher N and lower C:N ratios than conifers [40], [41], [42]. Our results showing lower soil N and increasing soil C:N ratios in stands with increasing conifer dominance are consistent with the interpretation that chemical differences in conifer litter inputs contribute to shifts in soil C and N chemistry.

The data also demonstrated a pattern of higher mineral nutrient availability in the surface soil horizons of aspen stands (Table 1). Due to similarity in soil texture across stand types, and the lack of stand differences in soil nutrients in the deeper B horizon, trends in soil chemistry that were only apparent in the surface soils (OA) are likely influenced by differences in litter inputs. Conifer species tend to have lower foliar mineral nutrient concentrations than deciduous species [43], suggesting that reductions in surface soil fertility with increasing conifer abundance may correspond to greater proportions of litter inputs from conifer species. Aspen understories also tend to have much higher productivity and greater biodiversity than conifer stands including N-fixing legumes [11], [12], which may also contribute to greater aspen stand fertility via increased soil nutrient inputs and cycling. Differences in soil pH can also influence differences in soil nutrient availability between aspen and conifer soils [44]. In other forest systems, conifers have been shown to lower soil pH [45]. However, we did not observe statistically significant differences in soil pH across our study gradient (Table 1), suggesting that the influence of conifers on soil chemistry in our study system may still be developing.

It has been suggested that lower soil C:N ratios in aspen dominated stands may explain differences in microbial community composition and nutrient cycling rates compared to conifer dominated stands [13], [15]. Microbial biomass can also be responsive to changes in forest composition [46]. However, a reciprocal transfer study of aspen soils and conifer soils found that the microbial biomass and community structure was unaffected by relocation to the contrasting forest stand type, suggesting that differences in C:N ratios in aspen-conifer forests may have a stronger influence on microbial activity than microbial community structure [14].

### Soil moisture content

Environmental influences on plant community responses in subalpine forests are often mediated through changes in soil moisture [47], [48]. In contrast to more mesic boreal systems, subalpine forests in the western U.S. often experience drier conditions toward the end of summer [47], [48] as water derived from snowpack disappears [49]. Consistent with these patterns, we observed a steady decline of soil moisture content, regardless of stand type, through the summer season (Figure 2). Soil moisture content differed markedly between aspen stands and meadow (21% VWC) and conifer stands (15% VWC) at the beginning of summer but converge by mid to late summer (Figure 2). Stand replacement of beech to spruce also yielded similar patterns of decreased soil moisture content in subalpine forests [50]. Differences between deciduous and evergreen species in canopy architecture and leaf persistence through winter result in aspen stands having significantly greater winter snowpack accumulation than conifer stands [17]. Convergence in soil moisture content between stand types by the end of summer may be partially driven by aspen stands having higher summer evapotranspiration rates than conifer stands [17], [51].

Soil texture and organic matter content play an important role in soil moisture storage and retention in surface soils. While we did not observe any differences in soil texture across stand types, aspen stands had higher soil organic matter content (Table 1), which increases water holding capacity of soils. Duff accumulation in conifer dominated stands exhibits significant water repellency and this may also have negative influences on water penetration and retention into the upper soil surface layers as conifer dominance increases [52].

### Biological activity of soils

Trends in soil respiration across stand type changed throughout the summer, indicating that abiotic factors likely have important influences on soil respiration rates (Figure 2). More favorable soil moisture and temperature conditions in aspen stands likely contribute to higher rates of respiration [19], [53]. Greater pools of organic carbon substrate, lower C:N ratios (Table 1), higher levels of microbial biomass and finer root biomass can also contribute to higher soil respiration rates [15]. Greater soil organic matter (which our data shows to be highest in aspen stands) would result in more substrate for microbial activity [19]. As discussed previously, aspen stands also had lower soil C:N ratios than conifer stands, which would tend to promote microbial decomposition contributing to greater CO_2_ efflux [53].

The observed July peak of soil respiration in all stand types is likely explained by optimal soil moisture and temperature conditions. The pattern suggests that total soil respiration is constrained by low temperatures in the early summer and soil moisture deficit toward the end of summer [54]. Drought in aspen forests has been shown to have negative effects on soil respiration rates by interfering with microbial metabolism, and reducing root respiration [48], [55]. Low soil moisture conditions can limit microbial acquisition of organic substrates and cause microbial dormancy [56]. While aspen stands had higher soil respiration across the entire summer, it was much more responsive to peak soil moisture content and temperature conditions (July) than meadow, mixed or conifer dominated stands (Figure 2). These results suggest that aspen soils are much more biologically active than the other soil types, particularly under optimal environmental conditions. These data suggest that shifts in canopy composition can significantly influence carbon sequestration dynamics via differences in organic matter accumulation and soil respiration rates.

### Plant community responses

These data indicate that higher aspen abundance in aspen-conifer forests [26] is related to greater soil resource availability and respiration. Changes in the abundance of soil resources are likely to have significant impacts on plant community development. As an example, changes in soil chemistry driven by conifers documented in this study have been shown to have stronger negative effects on primary metabolism, growth, and defense of establishing aspen than fir seedlings [57]. Furthermore, light limitation imposed by conifer expansion also constrains symbiotic mycorrhizal associations on aspen roots that can further limit their acquisition of soil nutrients [58]. Changes in disturbance regimes or climate scenarios that favor conifer expansion or loss of aspen are likely to decrease soil resource availability, with strong potential feedbacks on plant community development.
